# Oxidative Phosphorylation-Mediated E-Selectin Upregulation Is Associated With Endothelia–Monocyte Adhesion in Human Coronary Artery Endothelial Cells Treated With Sera From Patients With Kawasaki Disease

**DOI:** 10.3389/fped.2021.618267

**Published:** 2021-02-22

**Authors:** Danfeng Zhang, Lingjuan Liu, Yuxing Yuan, Tiewei Lv, Xupei Huang, Jie Tian

**Affiliations:** ^1^Department of Cardiology, Ministry of Education Key Laboratory of Child Development and Disorders, National Clinical Research Center for Child Health and Disorders, China International Science and Technology Cooperation Base of Child Development and Critical Disorders, Children's Hospital of Chongqing Medical University, Chongqing, China; ^2^Chongqing Key Laboratory of Pediatrics, Chongqing, China; ^3^Department of Biomedical Science, Charles E. Schmidt College of Medicine, Florida Atlantic University, Boca Raton, FL, United States

**Keywords:** Kawasaki disease, coronary artery lesion, oxidative phosphorylation, E-selectin, adhesion molecules

## Abstract

**Background and aims:** E-selectin is a cell adhesion molecule of the vascular endothelium that mediates leukocyte rolling in the early inflammatory responses in many diseases including Kawasaki disease (KD). Previous studies have demonstrated that the expression levels of E-selectin was significantly increased in the sera of KD patients and in endothelial cells of KD patient's autopsy. In this study, we aimed to examine E-selectin levels in endothelial cells treated with sera from KD patients and explore the underlying mechanisms.

**Methods:** Human coronary artery endothelial cells (HCAECs) were randomly incubated with sera from either healthy children [healthy control (HC group)] or pediatric KD patients [assigned as KD with coronary artery lesion (KD-CAL+ group) and KD without coronary artery lesion (KD-CAL– group)]. E-selectin levels were determined by RT-qPCR, Western blotting, and immunofluorescence. Cell adhesion assay was performed to quantify the role of E-selectin in intercellular adhesion. High-throughput cell RNA sequencing followed by functional validation was performed to explore the underlying mechanism.

**Results:** E-selectin levels were significantly increased in KD-CAL+ group vs. HC group and KD-CAL– group. Compared with the KD-CAL– group, endothelia–monocyte adhesion was increased in the KD-CAL+ group, while E-selectin-specific siRNA could significantly rescue it. High-throughput cell RNA sequencing analysis also found a significant difference in oxidative phosphorylation (OXPHOS) levels between KD-CAL+ group and KD-CAL– group. Functional validation results further confirmed that the OXPHOS was upregulated in the KD-CAL+ group and KD-CAL– group compared to that in the HC group, while the KD-CAL+ group exhibited a higher OXPHOS than the KD-CAL– group. We also found that the E-selectin levels and endothelia–monocyte adhesion were significantly decreased by OXPHOS inhibitor oligomycin in the KD-CAL+ group and KD-CAL– group, respectively.

**Conclusion:** Sera from KD patients stimulate OXPHOS levels and enhance E-selectin expression in HCAECs, which may contribute to the development of CAL in KD patients.

## Introduction

Kawasaki disease (KD) is an acute systemic vasculitis that was first described by Tomisaku (1). The coronary arteries are the most affected vessels (2), now the leading cause of acquired heart disease in developed countries (3). The incidence of coronary artery lesion (CAL) varies from regions and races (4) [19% in the UK (5), 3.2% in the USA (6), 3.5% in Canada (7), 15.9% in Shanghai (8), and 9.7% in Japan (9)]. Although the use of intravenous immunoglobulin (IVIG) has been shown to be effective in decreasing the incidence of CAL formation in the acute phase (10), the pathophysiological mechanism of CAL formation remains unknown. Orenstein et al. (11) demonstrated three interrelated processes of CAL formation in autopsy specimens from patients who died of KD with cardiovascular events. The studies showed that the infiltration of neutrophils in the vascular wall was stimulated by the damage of endothelial cells at an early stage, resulting in a dysfunction of vascular barrier functions (11, 12). The infiltration of inflammatory cells depends on adhesion molecules and selectin family, such as intercellular adhesion molecule (ICAM) and E-selectin, etc. (13).

Compared with the ICAM-1, E-selectin is a more effective molecule in slowing down leukocyte rolling *in vivo* and *in vitro* (14), which serves as the only selectin expressed by human endothelial cells during inflammatory stimulation *in vitro* and plays a more significant role in recruiting inflammatory cells in humans (15). The expression levels of E-selectin can be stimulated by a series of inflammatory molecules including tumor necrosis factor (TNF-α), interleukin-1 (IL-1), or bacterial lipopolysaccharide (LPS). Previous studies showed that E-selectin levels rose to peak after being stimulated by inflammatory cytokines for 4 h then gradually decreased *in vitro* (16). Moreover, E-selectin has been shown to be involved in rheumatoid arthritis (RA) and atherosclerosis (16, 17). A previous study found an increment of the soluble form of E-selectin (sE-selectin) in KD patients' sera in the acute phase (18), and the expression of E-selectin was also observed in the coronary arteries of KD patients with CAL (19), while a few studies focused on the relationship between E-selectin and inflammatory cell adhesion in CAL formation of KD and the underlying mechanism. Therefore, in the present study, we aimed to examine E-selectin levels in endothelial cells treated with KD patients' sera with or without CAL and explore the underlying mechanism involved in inflammatory cell adhesion of CAL.

## Methods

### Human Sample Collection

All subjects were from outpatient and inpatient departments of the Children's Hospital affiliated with Chongqing Medical University. All KD patients met the diagnostic criteria proposed by the Japanese Circulation Society Joint Working Group (JCS) in 2017. The clinical data of the subjects are presented in Table 1. The luminal dimensions of the coronary arteries were measured by echocardiography; KD-CAL was defined as Z score >2. During the acute phase, all KD patients were treated with IVIG (2 g/kg) and aspirin (30–50 mg/kg/day) initially. IVIG resistance was defined as sustained or recurrent fever (>38°C) at least 36 h after IVIG infusion. Sera from KD patients were collected before IVIG treatment, and sera from healthy children were collected as healthy control (HC). The study was approved by the Medical Research Ethics Committee of the Children's Hospital affiliated with Chongqing Medical University. Written informed consent was obtained from the parents of all subjects. All samples were preserved at −80°C.

**Table 1:** Characteristics of healthy controls and KD patients with different coronary artery status.

		**KD(40)**			
		**CAL+**	**CAL-**	**HC**	^*^P**-value**
Sex	Male, *n*	13	12	7	0.74
	Female, *n*	7	8	13	
Age, month		25	34.4	58.6	0.2
IVIG resistance, *n* (%)		2 (10%)	0	NA	0.15
WBC (×10^9^/L), median		15.3	13.4	8.7	0.11
PLT (×10^9^/L), median		401	396	307	0.26
HB (g/L), median		98	106	113	0.97
CRP (mg/L), median		45	37	NA	0.64
ESR (mm/h), median		74	56	NA	0.73

* *P-values were calculated by Man-Whitney U test for continuous variables and chi square test for categorical variables between CAL+ and CAL– group. KD, Kawasaki disease; CAL, coronary artery lesion; IVIG, intravenous immunoglobulin; CRP, C-reactive protein; ESR, erythrocyte sedimentation rate; WBC, white blood cell count; NA, not applicable*.

### Cell Culture

Human coronary artery endothelial cells (HCAECs) were cultured in Dulbecco's modified Eagle's medium (DMEM)/F12, supplemented with 10% fetal bovine serum (FBS), 50 units/ml penicillin, and streptomycin at 37°C in 5% CO_2_. Before performing subsequent experiments, cells were treated with DMEM/F12 supplemented with 15% sera from KD patients or HC for 4 h. THP-1 cells were cultured in RPMI-1640, supplemented with 10% FBS, 50 units/ml penicillin, and streptomycin at 37°C in 5% CO_2_. Cells at passages 4–6 were used for the following experiments *in vitro*.

### Total RNA/DNA Isolation and Quantitative Real-Time PCR Analysis

Total RNA was extracted with TRIzol reagent (Sigma). RNA purity and concentration were determined by measuring the absorbances at 260 and 280 nm, respectively. PrimeScript™ RT reagent kit (TaKaRa) was used to obtain complementary DNA (cDNA). The total genomic DNA was extracted with the animal tissue/cell genomic DNA extraction Kit (Solarbio). qPCR reactions in the presence of SYBR Green were performed on CFX 96™ Real-Time System (BIO-RAD). The primer sequences for qPCR were as follows: sele-For: *5*′ − *GCCTGCAATGTGGTTGAGTG* − 3′ and sele-Rev: *5*′ − *GCACCTCACAGAGCCATTCT* − 3′*;* GAPDH-For: *5*′ − *GTCTCCTCTGACTTCAACAGCG* − 3′ and GAPDH-Rev: *ACCACCCTGTTGCTGTAGCCAA-3*′*;* mtDNA-For: *5*′ − *GGGGAAGCAGATTTGGGTAC* − 3′ and mtDNA-Rev: *5*′ − *AGGGTGGGTAGGTTTGTTGG* − 3′*;* 18SRNA-For: *5*′ − *CAGGAAGGAAGGCTGGAAG* − 3′ and 18SRNA-Rev: *5*′ − *CGGGAAATCGTGCGTGAC* − 3′. The relative expression was calculated using the 2^−ΔΔCt^ method.

### High-Throughput RNA Sequencing

Preparation of cDNA libraries and Illumina novaseq 6000 RNA sequencing were performed at Novogene Co., LTD (Beijing, China). Differential expression analysis of HCAECs incubated with serum from acute KD patients with CAL (KD-CAL+, *n* = 3) or KD without CAL (KD-CAL–, *n* = 3) was performed using the DESeq2 R package (1.16.1). DESeq2 provides statistical routines for determining differential expression in digital gene expression data using a model based on the negative binomial distribution. Genes with a *P*-value <0.05 found by DESeq2 were assigned as differentially expressed.

Gene Ontology (GO) enrichment analysis of differentially expressed genes was implemented by the Cluster Profiler R package, in which gene length bias was corrected. GO terms with corrected *P*-value <0.05 were considered significantly enriched by differentially expressed genes.

The Cluster Profiler R package was used to test the statistical enrichment of differential expression genes in Kyoto Encyclopedia of Genes and Genomes (KEGG) pathways.

### Western Blotting Assays

Total fresh protein extracts were isolated from HCAECs using enhanced radioimmunoprecipitation assay (RIPA) lysis buffer (Beyotime) supplemented with protease and phosphatase inhibitors (Roche). Pierce BCA Protein Assay Kit (Thermo) was used for protein quantification. Equal protein samples (30–100 μg) were separated on 10% sodium dodecyl sulfate (SDS)-polyacrylamide gel electrophoresis (PAGE) gels. The following primary antibodies were used: rabbit anti-E-selectin (1:500 dilutions, CST, Danvers, USA), mouse anti-GAPDH (1:1,000 dilutions, OriGene, Beijing, China). Horseradish peroxidase-coupled secondary antibodies (1:5,000 dilutions, Proteintech, Wuhan, China) were used to detect primary antibody binding.

### Immunofluorescence Assays

HCAECs were fixed with 4% paraformaldehyde and sealed with bovine serum albumin (BSA). Fluorescein isothiocyanate (FITC)-labeled goat anti-rabbit IgG (H+L) secondary antibody (1:500 dilutions, Beyotime) was used to detect rabbit anti-E-selectin (1:200 dilutions, CST, Danvers, USA). Dil (Beyotime) was used for cell membrane labeling. Here, 4′,6-diamidino-2-phenylindole (DAPI; Keygen) was used for nucleus labeling. Fluorescence was measured by confocal microscopy. The NIS Element Viewer statistical software was used to calculate the fluorescence intensity.

### Endothelia-Monocyte Adhesion Assay

The HCAECs were seeded into 12-well plates, cultured to 100% fusion. THP-1 cells were incubated for 30 min at 37°C labeled with 2′,7′-bis-(2-carboxyethyl)-5-(and-6)-carboxyfluorescein acetoxymethyl ester (BCECF-AM). The fluorescently labeled THP-1 (1 × 10^5^/well) cells were added into HCAECs, then incubated at 37°C for 1 h. Suspended THP-1 cells were removed with Hank's balanced salt solution with HEPES buffer (HHBS), cell lysis buffer was added, and scraped. The images were collected under a fluorescence microscope. The fluorescence intensity was measured at microplate reader excitation and emission wavelengths of 490 and 530 nm, respectively.

### Measurement of ATP Content in Cells

ATP Assay Kit (Beyotime) was used to measure the content of ATP in HCAECs. Samples were prepared according to the instructions. The measurements were made with a chemiluminescence apparatus.

### Assessment of Mitochondrial Membrane Potential

Mitochondrial membrane potential assay kit with JC-1 fluorescent probe (Beyotime) was used to measure the mitochondrial membrane potential. Fluorescence was measured by confocal microscopy.

### Assessment of Mitochondrial Respiratory Chain Complex I Activity

Mitochondrial complex I activity detection assay kit (Solarbio) was used to measure the mitochondrial respiratory chain complex I activity. The absorbance was measured at 340 nm with a microplate reader. Record absorbance A1 at 10 s, reaction at 37°C for 2 min, record absorbance A2 at 2 min, calculate ΔA = A1–A2. Pierce BCA Protein Assay Kit (Thermo) was used for protein quantification. Complex I activity (U/mg prot) = [Δ*A*×*Vtotal*÷(ε× *d*) ×10^9^] ÷ (Vsample × Cpr) ÷ T = 2,679.5 × ΔA ÷ Cpr (Vtotal = 2 × 10^−4^ L; ε: NADH molar extinction coefficient, 6.22 × 10^3^ L/mol/cm; d: 96-well plates diameter, 0.6 cm; Vsample = 0.01 ml; T: Reaction time, 2 min; Cpr: Sample protein concentration, mg/ml).

### Statistical Analysis

All values were expressed as means ± SEM. Mann–Whitney U test or ANOVA followed by the Bonferroni *post-hoc* test was used to analyze differences among groups. *P* < 0.05 was considered statistically significant.

## Results

### Kawasaki Disease Sera Increased E-Selectin Level in Human Coronary Artery Endothelial Cells

**Figure 1:**
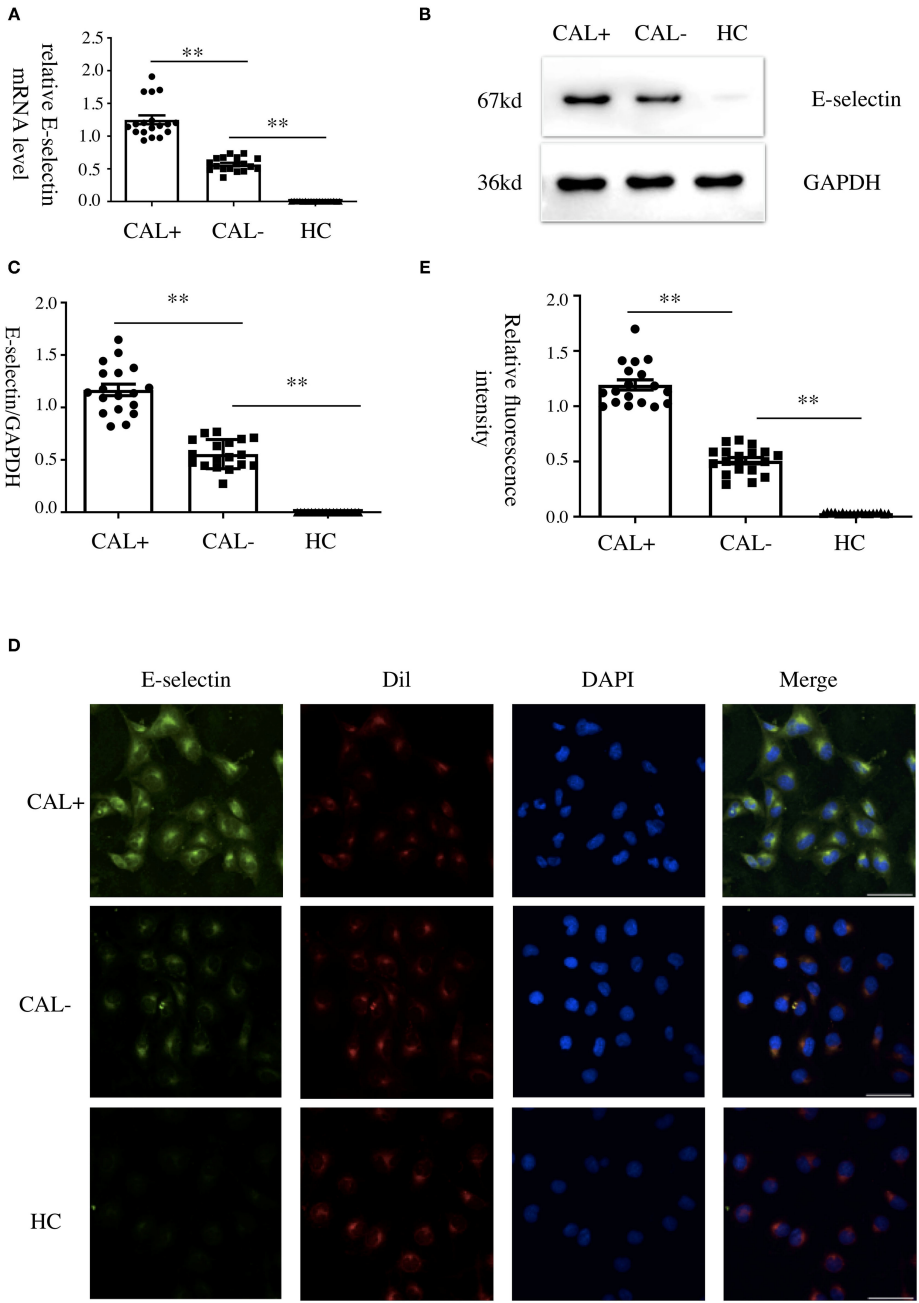
Kawasaki disease (KD) sera increased E-selectin level in human coronary artery endothelial cells (HCAECs). **(A–C)** HCAECs were treated with medium containing 15% sera from healthy controls (HCs; *n* = 6), KD without coronary artery lesion (KD-CAL–; *n* = 6), and KD with coronary artery lesion (KD-CAL+; *n* = 6) for 4 h. The E-selectin mRNA levels were measured by qPCR (**A**, HC = 6, KD-CAL– = 6, KD-CAL+ = 6), and protein levels were measured by Western blot (**B**, HC = 6, KD-CAL– = 6, KD-CAL+ = 6). **(C)** Statistical analysis of immunoblots. **(D,E)** HCAECs were treated with medium containing 15% sera from HCs (*n* = 6), KD-CAL– (*n* = 6), and KD-CAL+ (*n* = 6) for 4 h; localization and expression of E-selectin (green) in HCAECs were measured by immunofluorescence. **(D,E)** Statistical analysis of immunofluorescence. The bar graphs are mean ± SEM. ^**^P < 0.01 compared with the indicated group.

We first detected the levels of E-selectin in HCAECs after treatment with KD sera or healthy control sera for 4 h. As shown in Figures 1A–C, incubation of HCAECs with sera from KD subjects with CAL+ resulted in a significantly higher expression of E-selectin when compared with CAL– and HC group, which followed the order of HC < KD-CAL– <KD-CAL+. Intracellular localization and the expression levels of E-selectin were determined by immunofluorescence. As shown in Figures 1D,E, E-selectin was mainly distributed in the cell membrane and cytoplasm when treated with serum from KD subjects. The expression levels of E-selectin were consistent with Western blot and qPCR.

### E-Selectin Was Involved in Kawasaki Disease Endothelia–Monocyte Interaction

**Figure 2:**
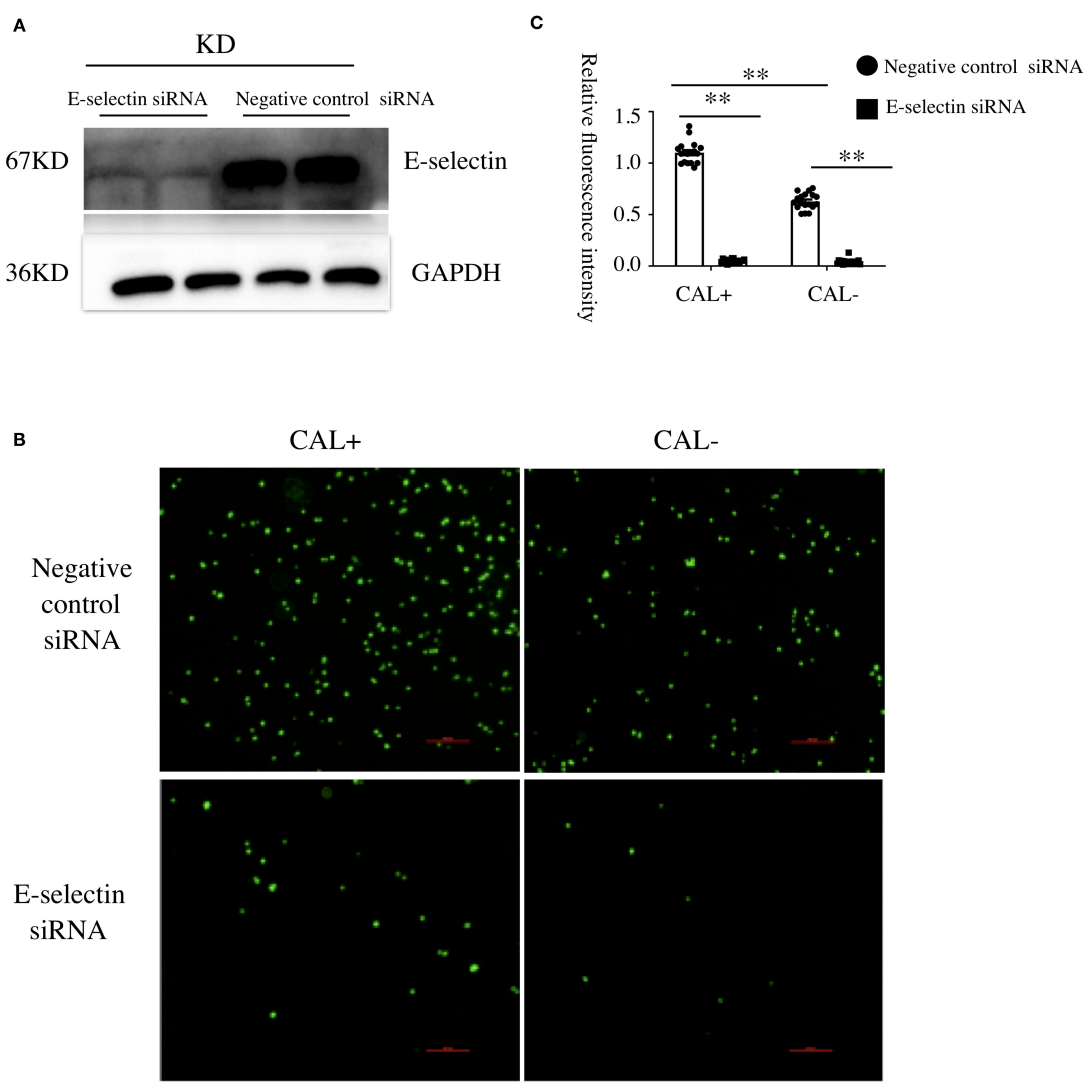
E-selectin was involved in Kawasaki disease (KD) endothelia–monocyte interactions. Human coronary artery endothelial cells (HCAECs) were treated with specific E-selectin siRNA (100 nM) or negative control siRNA (100 nM) for 72 h, followed by medium containing 15% sera from KD-CAL– (*n* = 6) and KD-CAL+ (*n* = 6) subjects for 4 h. Changed the medium to Hank's balanced salt solution with HEPES buffer (HHBS) and incubated with 2′,7′-bis-(2-carboxyethyl)-5-(and-6)-carboxyfluorescein acetoxymethyl ester (BCECF-AM)-labeled THP-1 cells for 1 h, then the suspended cells were washed with phosphate buffered saline (PBS). **(A)** The effects of siRNA (pooled from three KD subjects). **(B)** Representative images used quantification of THP-1 cells (green, BCECF-AM) attached to human umbilical vein endothelial cells (HUVECs). **(C)** Quantitative analysis of adherent THP-1 cells lysate fluorescence at 490-nm excitation and 530-nm emission. All samples were normalized to HCAECs with CAL serum treatment. The bar graphs are mean ± SEM. ^**^P < 0.01 compared with the indicated group.

To investigate the role of E-selectin in endothelia–monocyte interactions in KD, we performed cell adhesion assays. HCAECs were pretreated with specific siRNA of E-selectin (sense: 5′-GGUUGAAUGCACCACUCAATT-3′ and antisense: 5′-UUGAGUGGUGCAUUCAACCTT-3′) or negative control siRNA (sense: 5′-UUCUCCGAACGUGUCACGUTT-3′ and antisense: 5′-ACGUGACACGUUCGGAGAATT-3′) for 72 h before culturing with sera of KD subjects with CAL or without CAL for another 4 h, incubating with BCECF-AM-labeled THP-1 cells for 1 h. As shown in Figures 2A–C, the adhesion of THP-1 cells was significantly increased in the CAL+ group compared with the CAL– group, while this process was significantly rescued by siRNA of E-selectin.

### Upregulation of Oxidative Phosphorylation in Human Coronary Artery Endothelial Cells Treated With Kawasaki Disease With Coronary Artery Lesion Sera

**Figure 3:**
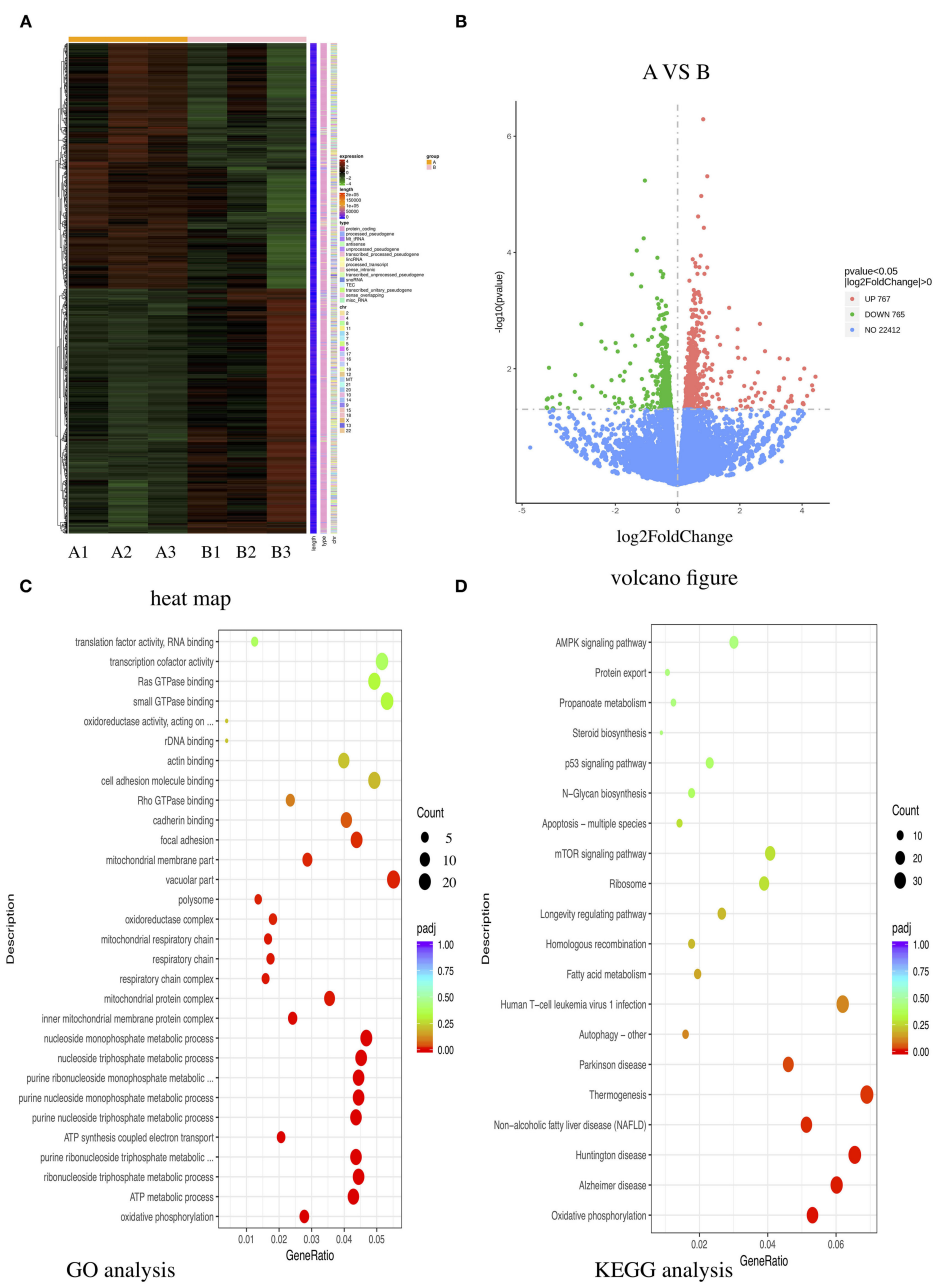
High-throughput RNA sequencing of Kawasaki disease (KD) sera-treated human coronary artery endothelial cells (HCAECs). HCAECs were treated with medium containing 15% sera from KD with coronary artery lesion (KD-CAL+; *n* = 3; group A) or KD without coronary artery lesion (KD-CAL–; *n* = 3; group B) for 4 h. The total RNA was isolated to perform Illumina novaseq 6000 RNA sequencing. **(A)** Differential expression gene clustering heat map. The abscissa is the sample name, and the ordinate is the normalized value of the differentially expressed gene FPKM. The redder the color, the higher the expression; the greener, the lower the expression. **(B)** Differential gene volcano-gram. The abscissa is log2FoldChange value, and the ordinate is –log10 padj or –log10 *p*-value. The blue dotted line represents the threshold line of the differential gene screening criteria. **(C)** Gene Ontology (GO) enrichment analysis bubble chart. The ordinate is GO Term, abscissa for the GO Term enrichment significant. **(D)** Kyoto Encyclopedia of Genes and Genomes (KEGG) enrichment analysis bubble chart. The ordinate is the KEGG pathway, and the abscissa is the significance level of the enrichment of the pathway.

**Figure 4:**
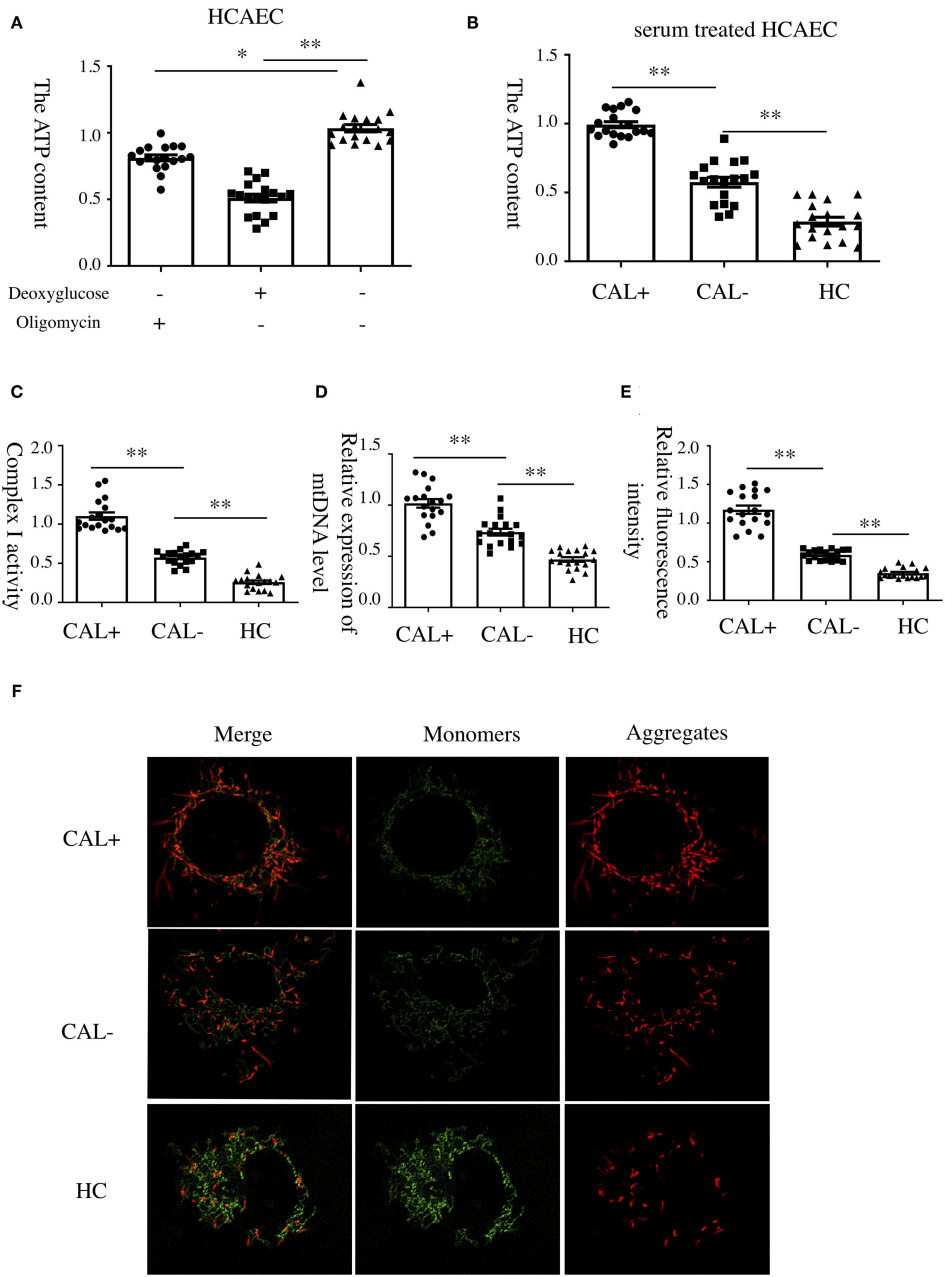
Increased oxidative phosphorylation in Kawasaki disease with coronary artery lesion (KD-CAL+) sera-treated human coronary artery endothelial cells (HCAECs). **(A)** HCAECs were treated with deoxyglucose (50 μg/ml) and oligomycin (10 mM) for 12 h. Intracellular ATP content was determined by chemiluminescence apparatus. **(B)** HCAECs were treated with medium containing 15% sera from healthy controls (HCs; *n* = 6), Kawasaki disease without coronary artery lesion (KD-CAL–; *n* = 6), and KD-CAL+ (*n* = 6) for 4 h. Intracellular ATP content was determined by chemiluminescence apparatus. **(C)** HCAECs were treated with medium containing 15% serum from HC (*n* = 6), KD-CAL– (*n* = 6), and KD-CAL+ (*n* = 6) for 4 h. Mitochondrial Complex I activity was determined in 340 nm with a microplate reader. **(D)** HCAECs were treated with medium containing 15% sera from HC (*n* = 6), KD-CAL– (*n* = 6), and KD-CAL+ (*n* = 6) for 4 h. Mitochondrial gene copy number was measured by qPCR. **(E,F)** HCAECs were treated with medium containing 15% sera from HC (*n* = 6), KD-CAL– (*n* = 6), and KD-CAL+ (*n* = 6) for 4 h. Mitochondrial membrane potential was determined by JC-1 (monomers green, aggregates red) analyzed by confocal microscopy. **(F)** Statistical analysis of relative fluorescence intensity (red/green). All samples were normalized to HCAECs with CAL serum treatment or control. The bar graphs are mean ± SEM. ^*^P < 0.05 and ^**^P < 0.01 compared with the indicated group.

To investigate the underlying mechanism of E-selectin upregulation in KD sera-treated HCAECs, high-throughput RNA sequencing of HCAECs was conducted, which were treated by sera from acute KD patients with or without CAL for 4 h. After that, the differential gene analysis including GO and KEGG enrichment analysis of differentially expressed genes was performed (PRJNA686838). As shown in Figures 3A,B, there were 767 genes upregulated and 765 genes downregulated compared with those of the KD-CAL- group. As shown in Figures 3C,D, genes associated with oxidative phosphorylation (OXPHOS) (Supplementary Table 1) and ATP metabolism (Supplementary Table 2) were significantly upregulated. Pathways associated with OXPHOS were significantly upregulated. To confirm the upregulated OXPHOS process, we first examined the ATP level of HCAECs treated with KD and HC sera because OXPHOS is the main way of ATP production. As shown in Figure 4B, HCAECs treated with sera from KD-CAL+ showed a high level of ATP content when compared to that of the KD-CAL– group and HC group. But previous studies have indicated that the main source of ATP in HCAECs was glycolysis, not OXPHOS (20). We also found that the ATP was significantly decreased in HCAECs after intervention with deoxyglucose (10 mM) for 12 h than that after oligomycin (50 μg/ml) (Figure 4A). To determine whether the increased ATP content was due to the upregulation of OXPHOS, mitochondrial membrane potential, mitochondrial gene transcription level, and mitochondrial complex I activity were tested. As shown in Figures 4C–F, HCAECs treated with serum from KD-CAL+ showed a high level of mitochondrial membrane potential, mitochondrial gene transcription level, and mitochondrial complex I activity compared with those of the KD-CAL– group and HC group.

### Oxidative Phosphorylation Was Involved in the Expression of E-Selectin and Endothelia–Monocyte Interaction

**Figure 5:**
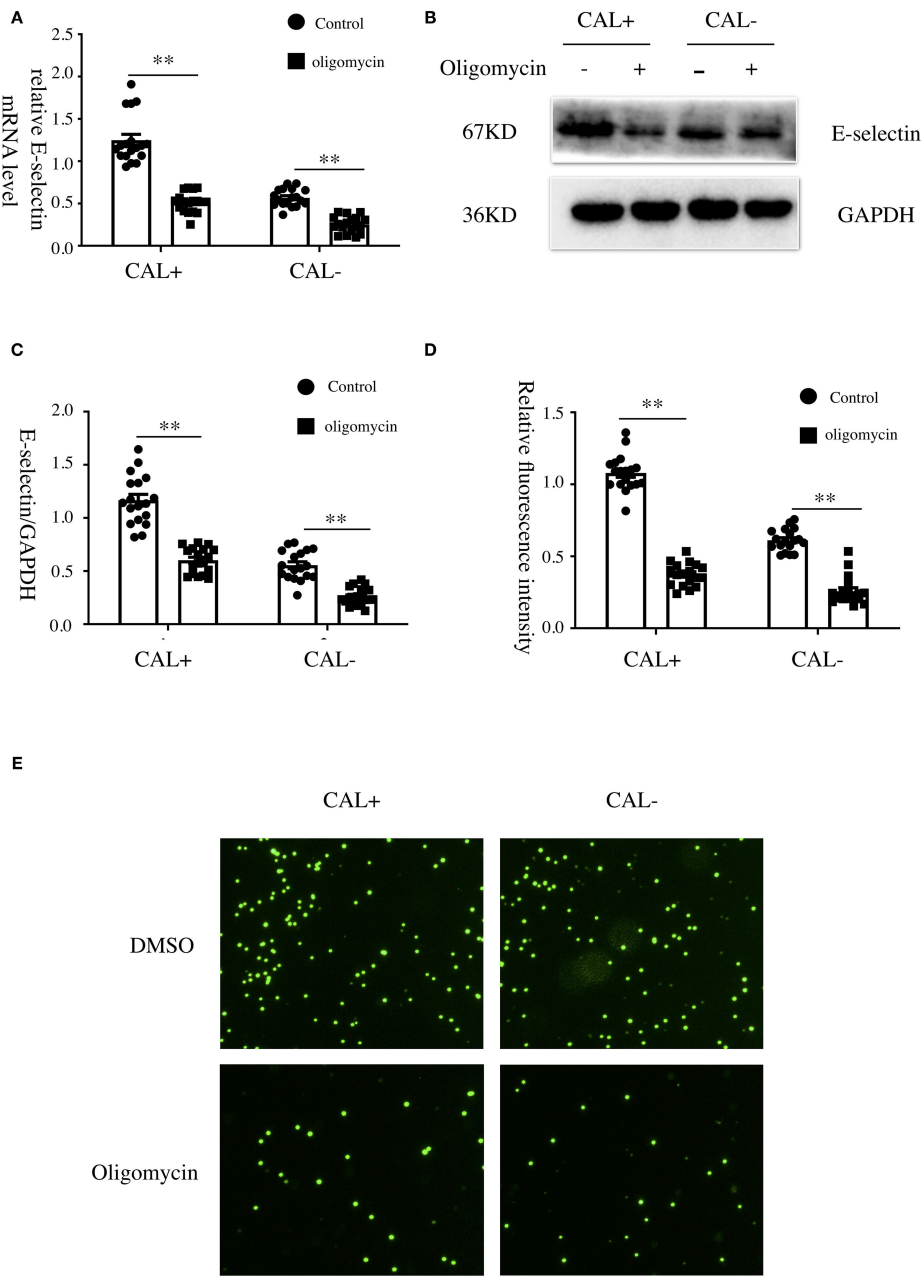
Oxidative phosphorylation was involved in the expression of E-selectin and monocyte–endothelial cell interaction. **(A–E)** Human coronary artery endothelial cells (HCAECs) were treated with medium containing 15% sera from Kawasaki disease without coronary artery lesion (KD-CAL–; *n* = 6) and Kawasaki disease with coronary artery lesion (KD-CAL+; *n* = 6) subjects plus oligomycin (50 μg/ml) or DMSO (same volume) for 4 h. The E-selectin mRNA levels were measured by qPCR **(A)**, and protein levels were measured by Western blot **(B)**. **(C)** Statistical analysis of immunoblots. **(E)** HCAECs were treated with medium containing 15% sera from KD-CAL– (*n* = 6) and KD-CAL+ (*n* = 6) subjects plus oligomycin (50 μg/ml) or DMSO (same volume) for 4 h then co-incubated with 2′,7′-bis-(2-carboxyethyl)-5-(and-6)-carboxyfluorescein acetoxymethyl ester (BCECF-AM)-labeled THP-1 cells for 1 h. Representative images used quantification of THP-1 cells (green) attached to human umbilical vein endothelial cells (HUVECs). **(D)** Quantitative analysis of adherent THP-1 cells lysate fluorescence at 490-nm excitation and 530-nm emission. All samples were normalized to HCAECs with CAL serum treatment. The bar graphs are mean ± SEM. ^**^P < 0.01 compared with the indicated group.

To explore the role of OXPHOS in the expression of E-selectin, oligomycin (50 μg/ml), a phosphorylation-specific inhibitor, or DMSO was added when HCAECs were treated with sera from KD patients with different coronary artery outcomes for 4 h. As shown, the expression levels of E-selectin mRNA and protein were significantly decreased after treated with oligomycin (Figures 5A–C). To examine the role of OXPHOS on intercellular adhesion, HCAECs were treated with sera from KD patients with different coronary artery outcomes with oligomycin (50 μg/ml) or DMSO for 4 h, then incubated with BCECF-AM-labeled THP-1 for another 1 h, and the adherent THP-1 cells were measured with a microplate reader. Cell adhesion was significantly reduced when treated with oligomycin (Figures 5D,E).

## Discussion

The main finding of this study is that the upregulation of E-selectin was related to the endothelia–monocyte interaction in KD sera-treated HCAECs, which may be one of the mechanisms of CAL formation. The upregulation of ATP production and mitochondrial OXPHOS may be the underlying mechanism.

E-selectin serves as an important link between endothelial cells and inflammatory cells. Elevated cellular E-selectin can be seen in some chronic inflammatory diseases, such as diabetes, atherosclerosis, and obesity (21–23). A previous study reported that the concentration of sE-selectin is directly correlated with cell surface expression (24). Due to the elevated expression of sE-selectin in KD patients (18), we hypothesized that E-selectin may be involved in the KD CAL process. In the present study, we found that KD-CAL+ sera-treated HCAECs exhibited a higher mRNA level and protein level of E-selectin when compared with KD-CAL– group and HC control. The levels of E-selectin were increased in RNA level in this study, and the increase in protein levels were consistent with the change in RNA level, basically excluding the possible false positive caused by sE-selectin in sera. E-selectin is mainly involved in the adhesion process of inflammatory cells; we found that the adherent THP-1 cells of the KD-CAL+ sera-treated HCAECs were significantly increased, which may lead to more severe inflammatory cell infiltration compared to that in the KD-CAL– group. The results indicated that a higher concentration of E-selectin may lead to more serious endothelia–monocyte interaction, which may be related to CAL formation, while ICAM and other molecules might be involved as well. To confirm the role of E-selectin in the adhesion process, we used specific E-selectin siRNA to silence the expression of E-selectin. As a result, the adhesion number of THP-1 cells was significantly decreased compared with negative control siRNA, indicating that the lack of the E-selectin may reduce the endothelia–monocyte interaction, which may be involved in the formation of CAL.

Rowley's team performed RNA sequencing using the coronary artery tissue of KD with KD-CAL (25). The results showed that T lymphocyte activation, antigen presentation and dendritic cell function, immunoglobulin production, and type I interferon response were the main significantly upregulated molecular pathways. However, in this study, the OXPHOS was significantly upregulated. The differences between the two studies may be complicated. Firstly, sources of samples are different, Rowley's study examined the total human coronary artery, the cell types are various, while the present study mainly focused on HCAECs and just *in vitro*. Secondly, we compared the differences between the KD-CAL+ group, KD-CAL– group, and HC group, while Rowley's study only focused on the KD-CAL+ group and HC group. Moreover, Rowley's study focused on the subacute or chronic stage of CAL, while the present study mainly focused on the acute stage. Although different conclusions have been drawn, all of them could be the potential molecular biological mechanism of KD CAL formation.

Mitochondria are the sites of OXPHOS involved in the regulation of inflammatory stimulating in many diseases (26–28). ATP in endothelial cells is mostly provided by glycolysis rather than OXPHOS (20, 29). As a result, the function of OXPHOS is neglected. Mitochondria play a role in endothelial inflammatory stimulation mainly by regulating the production of nitric oxide (NO) (30) and reactive oxygen species (ROS) (31), as well as the dynamics of intracellular [Ca^2+^] signaling (32). A previous study showed that ROS production plays an important role in coronary dysfunction in KD (33). In our study, the OXPHOS in HCAECs was stimulated by KD sera compared with HC sera, accompanied by the increment in the CAL+ group compared with the CAL– group, indicating that there may be a large number of ROS produced. A previous study reported that OXPHOS can regulate nuclear factor (NF)-κb signaling pathway in tumor necrosis factor (TNF)-α-stimulated endothelial inflammation (34). The expression of E-selectin is mainly dependent on the NF-κb signal pathway (35). In a previous study, OXPHOS uncouplers can reduce the expression of adhesion molecules through the NF-κB signaling pathway both *in vitro* and *in vivo* (36, 37). NF-κb signaling pathway is important in KD, IVIG can significantly inhibit the activation of NF-κb signaling pathway to reducing the incidence of CAL (38). In the present study, OXPHOS inhibitor can alleviate the expression of E-selectin, suggesting that the reduction of the ROS production and NF-κb pathway may be involved in the underlying mechanism of protecting endothelia from inflammation.

The limitation of this study is that we did not further explore the advancing mechanisms of how OXPHOS affects E-selectin expression and which specific factors in sera affect the expression of E-selectin. We also lack *in vivo* data to support our conclusions. Although freshly harvested endothelial cells isolated from the coronary arteries can most intuitively reflect the changes of the cell functions in patients during the development of KD, a measurement of *Lactobacillus casei* cell wall extract (LCWE)-induced mouse models in exploring the effects of E-selectin and OXPHOS pathway on vasculitis may warrant further studies.

In conclusion, the E-selectin and OXPHOS pathway reported in this study may be important factors involved in endothelia–monocyte interaction of CAL development in KD patients and may be useful clues as potential targets in the treatment of the disease. Further *in vivo* experiments are needed to confirm the conclusion.

## Data Availability Statement

The datasets presented in this study can be found in the NCBI SRA database, accession number: ID:PRJNA686838.

## Author Contributions

DZ contributed to conception, methodology, research conducte, data curation, and wrote the first draft of the manuscript. All authors contributed to manuscript revision, read, and approved the submitted version.

## Conflict of Interest

The authors declare that the research was conducted in the absence of any commercial or financial relationships that could be construed as a potential conflict of interest.
