# Supplementary material for: Oxidative Phosphorylation-Mediated E-Selectin Upregulation Is Associated With Endothelia–Monocyte Adhesion in Human Coronary Artery Endothelial Cells Treated With Sera From Patients With Kawasaki Disease
Source: Front Pediatr. 2021 Feb 22;9:618267. doi: 10.3389/fped.2021.618267 (PMC7937974; doi:10.3389/fped.2021.618267)
Supplement: Supplementary file 1 [file Table_1.DOCX]

| Gene | Log2 Fold Change | P Value |
| --- | --- | --- |
| COX8A | 0.75984217 | 1.07E-05 |
| ATP5MG | 0.56738722 | 0.00015155 |
| ATP5ME | 0.9517804 | 0.00018133 |
| NDUFA2 | 0.52611304 | 0.01342714 |
| COX6A1 | 0.93586659 | 0.03839865 |
| NDUFB8 | 0.56972067 | 0.04875328 |

Supplementary Table I:Oxidative phosphorylation related genes differentially expressed in HCAECs treated with sera from KD with CAL

| Gene | Log2 Fold Change | P Value |
| --- | --- | --- |
| COX8A | 0.75984217 | 1.07E-05 |
| ALDOA | -0.650082159 | 0.000123299 |
| ATP5MG | 0.56738722 | 0.00015155 |
| ATP5ME | 0.951780397 | 0.000181331 |
| ATP5IF1 | 0.63721189 | 0.00023324 |
| UQCC2 | 0.611102451 | 0.000495491 |
| COA6 | 0.594701812 | 0.000967211 |
| CYCS | 0.515111402 | 0.00123888 |
| MT-ATP8 | -1.924687347 | 0.005055713 |
| UQCC3 | 0.519844231 | 0.019438229 |
| TEFM | 0.577515201 | 0.020063401 |
| TMSB4X | 0.599649095 | 0.003920914 |
| MT-ND5 | -0.985442878 | 0.041530699 |
| NDUFA2 | 0.52611304 | 0.01342714 |
| COX6A1 | 0.93586659 | 0.03839865 |
| NDUFB8 | 0.56972067 | 0.04875328 |

Supplementary Table II: ATP metabolism related genes differentially expressed in HCAECs treated with sera from KD with CAL
